# Supplementary material for: Selective Interactions of O-Methylated Flavonoid Natural Products with Human Monoamine Oxidase-A and -B
Source: Molecules. 2020 Nov 17;25(22):5358. doi: 10.3390/molecules25225358 (PMC7697615; doi:10.3390/molecules25225358)
Supplement: Supplementary file 1 [file molecules-25-05358-s001.pdf]

SUPPORTING INFORMATION

Selective interactions of O-methylated flavonoid natural products with human monoamine oxidase-A and -B

Narayan D. Chaurasiya[1,3,#], Jacob Midiwo[2], Pankaj Pandey[3,4], Regina N. Bwire[5]
Robert J. Doerksen[4], Ilias Muhammad[3*] and Babu L Tekwani[1,3,#*]

[1] Department of Infectious Diseases, Division of Drug Discovery, Southern Research, Birmingham, AL 35205, USA; nchaurasiya@southernresearch.org (N.D.C)

[2] Department of Chemistry, University of Nairobi, P.O. Box 30197-00100, Nairobi, Kenya; jmidiwo@uonbi.ac.ke (J.M)

[3] National Center for Natural Products Research, Research Institute of Pharmaceutical Sciences, School of Pharmacy, University of Mississippi, University, MS 38677, USA; ppandey@olemiss.edu (P.P)

[4] Department of BioMolecular Sciences, Division of Medicinal Chemistry, Research Institute of Pharmaceutical Sciences, School of Pharmacy, University of Mississippi, University, MS 38677, USA; rjd@olemiss.edu (R.J.D)

[5] Department of pure and applied Chemistry, Masinde Muliro University of Science and Technology, P.O. Box 190, Postcode, 50100, Kakamega, Kenya; rbwire@mmust.ac.ke

[#] Present address: N.D.C[1] and B.L.T[1]

* Correspondance: btekwani@southernresearch.org (B.L.T); milias@olemiss.edu (I.M)
Tel: +1-205-581-2205 (B.L.T); +1-662-915-1051 (I.M)

TABLE OF CONTENT

Number	Details
SI 1	^{1}H NMR spectrum of compound **1** (3,4′-di-*O*-methylkaempferol)
SI 2	^{13}C NMR spectrum of compound **1**
SI 3	^{1}H NMR spectrum of compound **2** (2′-hydroxy-4′,6′-dimethoxy-chalcone)
SI 4	^{13}C NMR spectrum of compound **2**
SI,5	^{1}H NMR spectrum of compound **3** (2′,4′-dihydroxy-6′-methoxy-chalcone)
SI 6	^{13}C NMR spectrum of compound **3**
SI 7	^{1}H NMR spectrum of compound **4** (8-demethylsideroxylin)
SI 8	^{13}C NMR spectrum of compound **4**
SI 9	2D NMR COSY spectrum of compound **4**
SI 10	2D NMR HMQC spectrum of compound **4**
SI 11	2D NMR HMBC spectrum of compound **4**
SI 12	^{1}H NMR spectrum of compound **5** (4′-*O*-methylkaempferol)
SI 13	^{13}C NMR spectrum of compound **5**
SI 14	^{1}H NMR spectrum of compound **6** (5,7-dihydroxy-2',3',4',5'-tetramethoxyflavone)
SI 15	^{13}C NMR spectrum of compound **6**

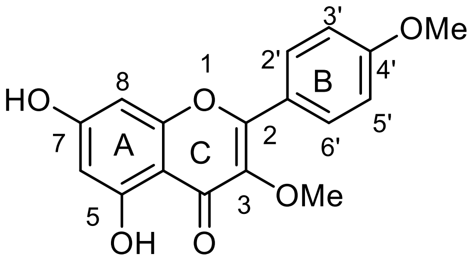

1

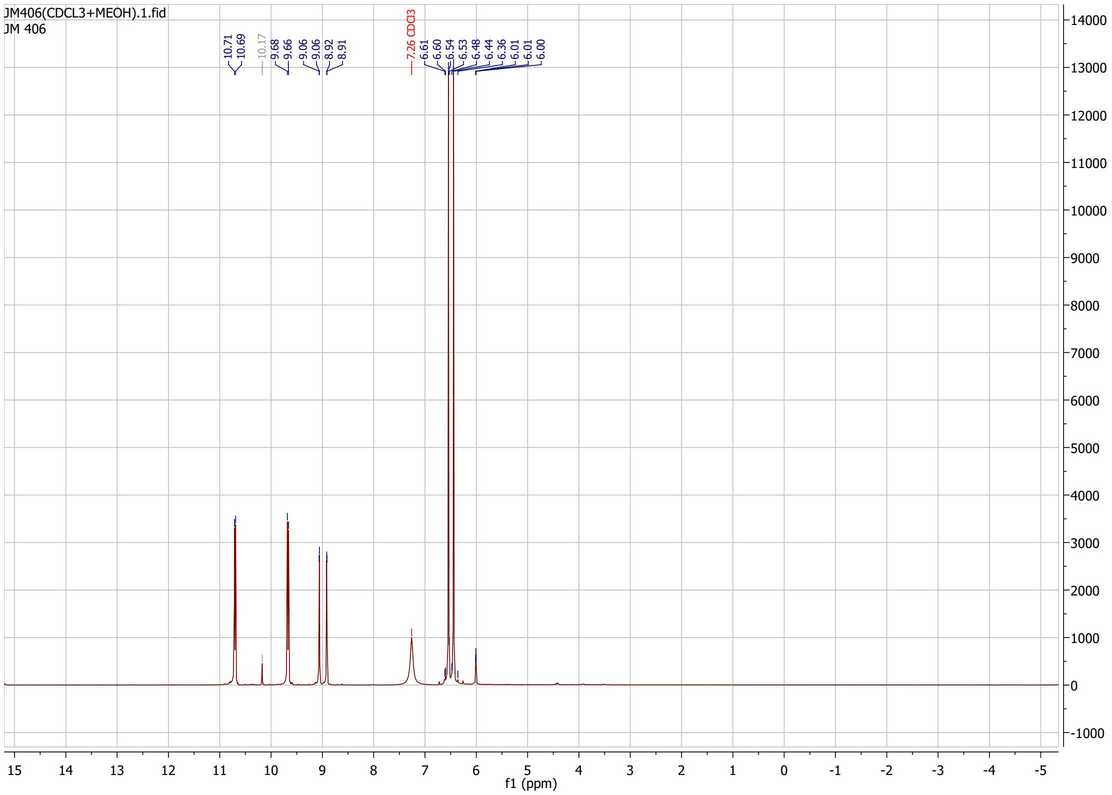

SI 1: ^{1}H NMR spectrum of compound **1** (500 MHz, $CDCl_3$+CD_3OD)

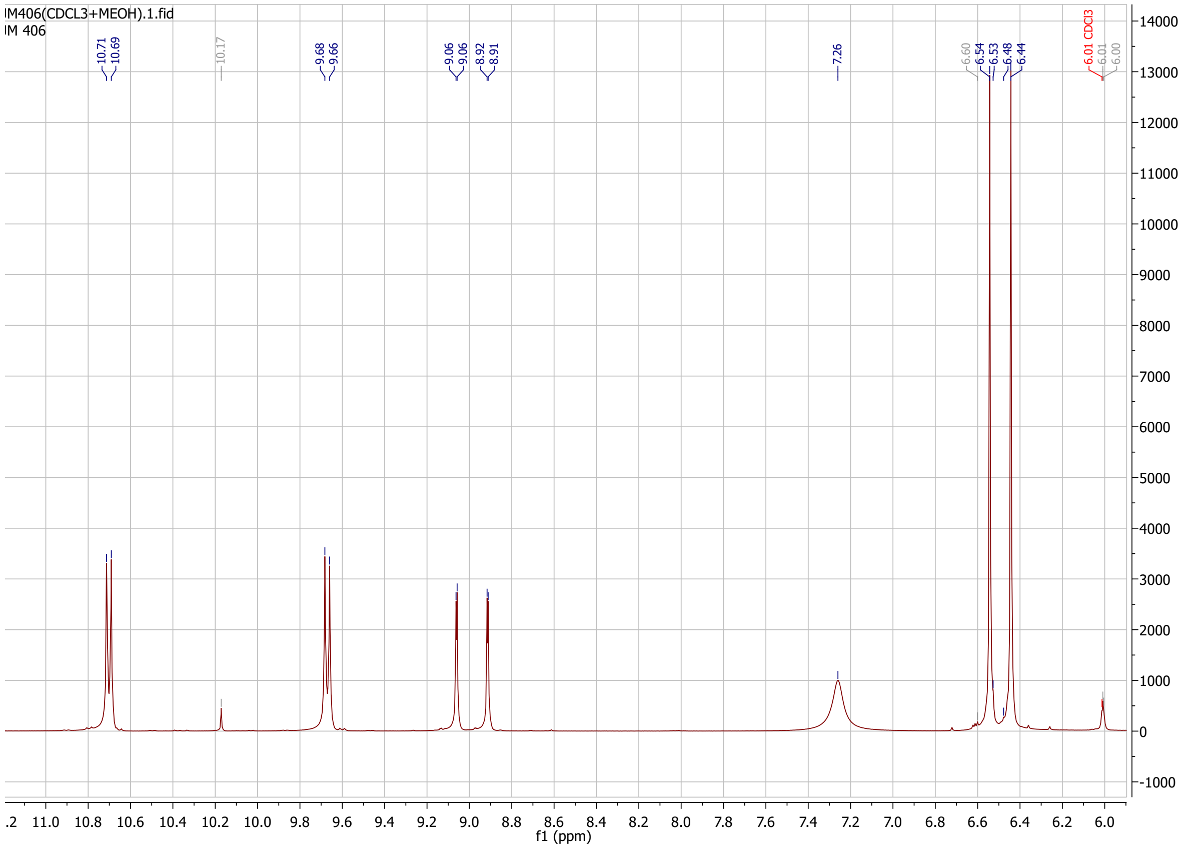

SI 1: ^{1}H NMR spectrum of compound **1** (500 MHz, $CDCl_3$+CD_3OD)

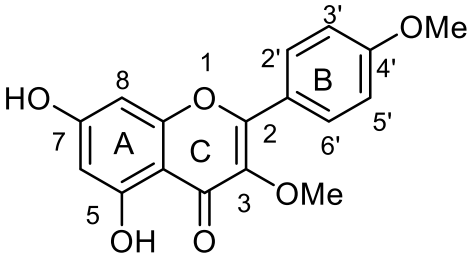

1

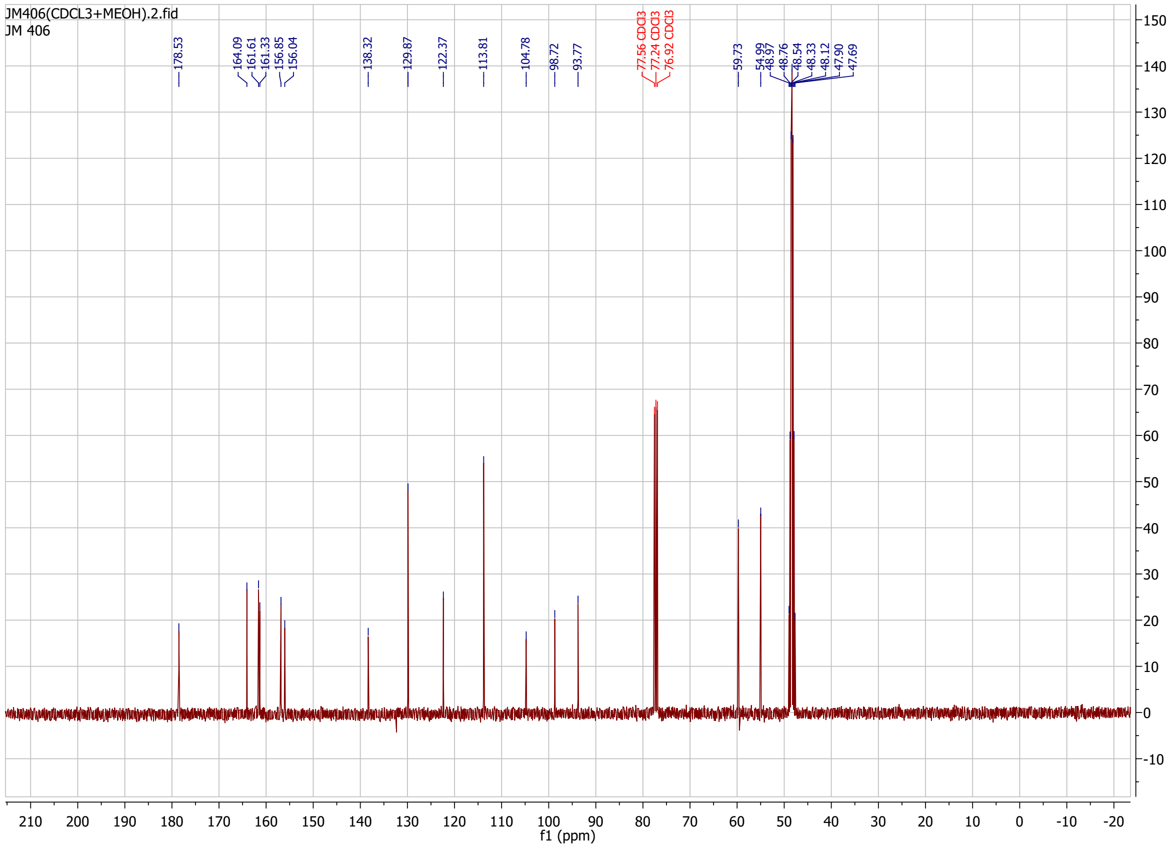

SI 2: ^{13}C NMR spectrum of compound **1** (125 MHz, $CDCl_3$+CD_3OD)

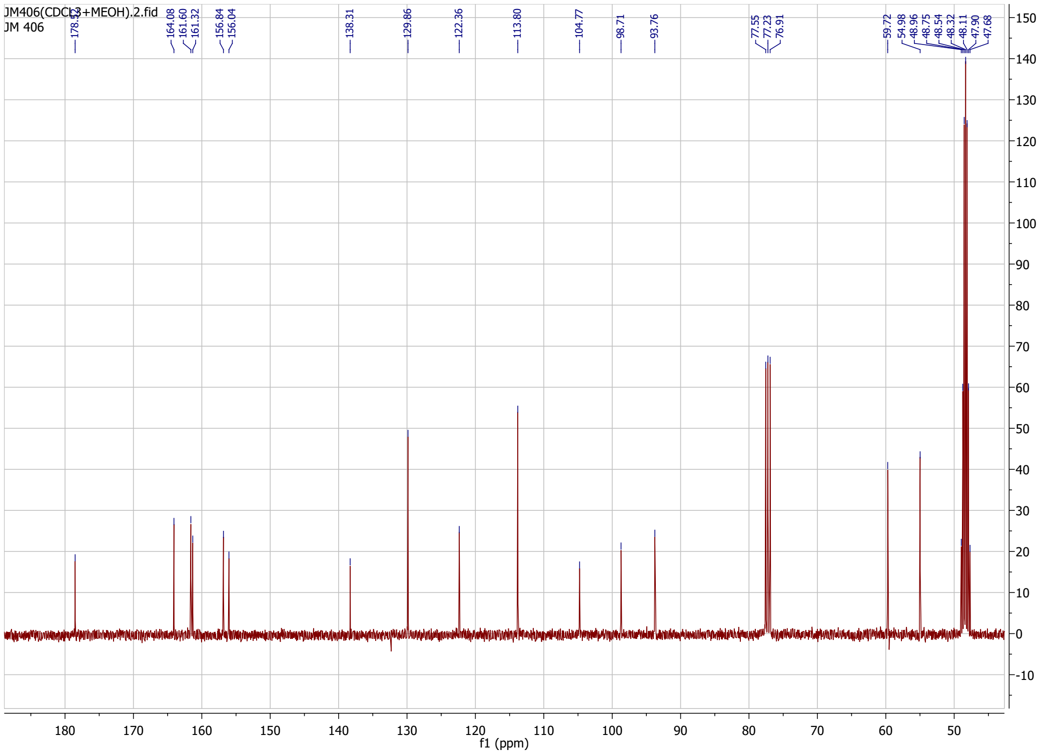

SI 2: ^{13}C NMR spectrum of compound **1** (125 MHz, $CDCl_3$+CD_3OD)

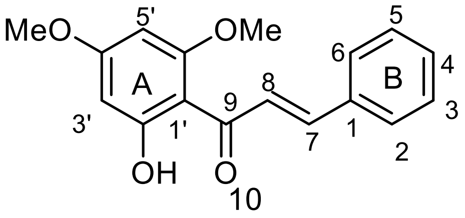

2

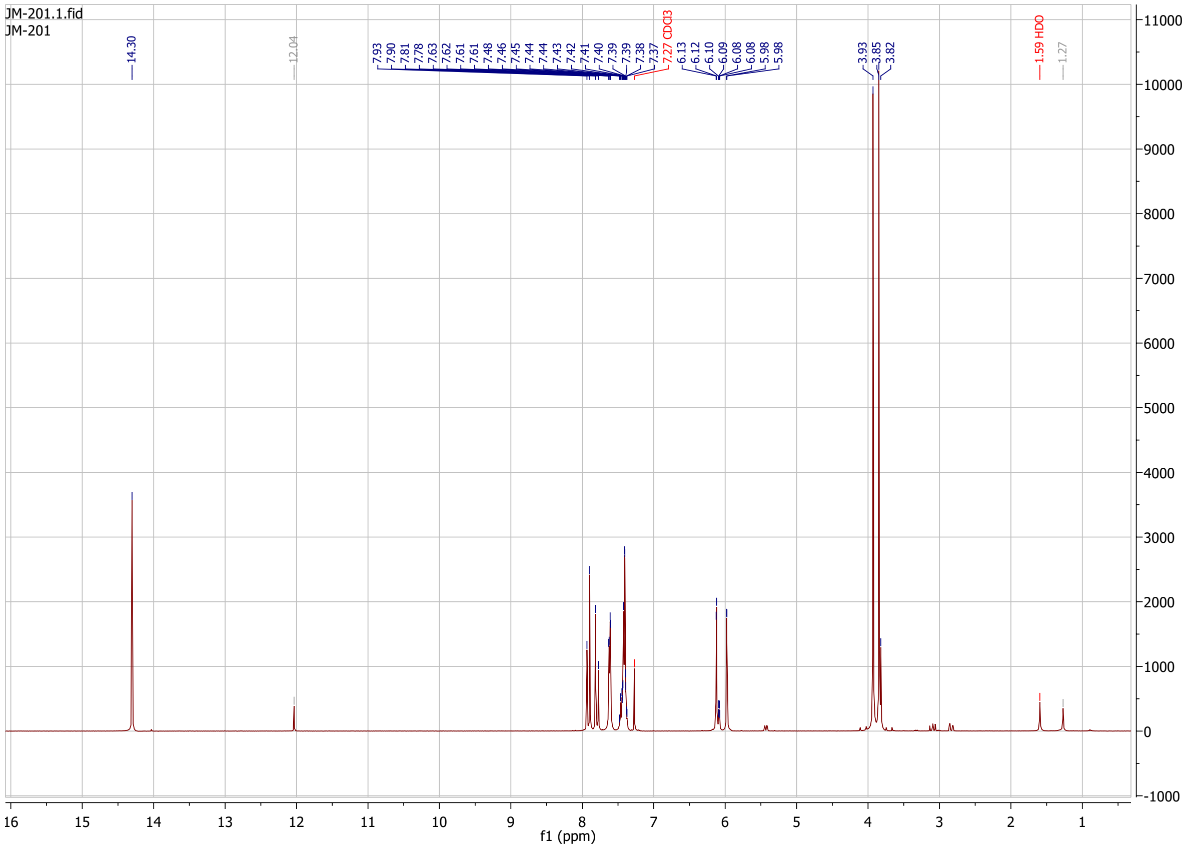

SI 3: ^{1}H NMR spectrum of compound **2** (500 MHz, $CDCl_3$)

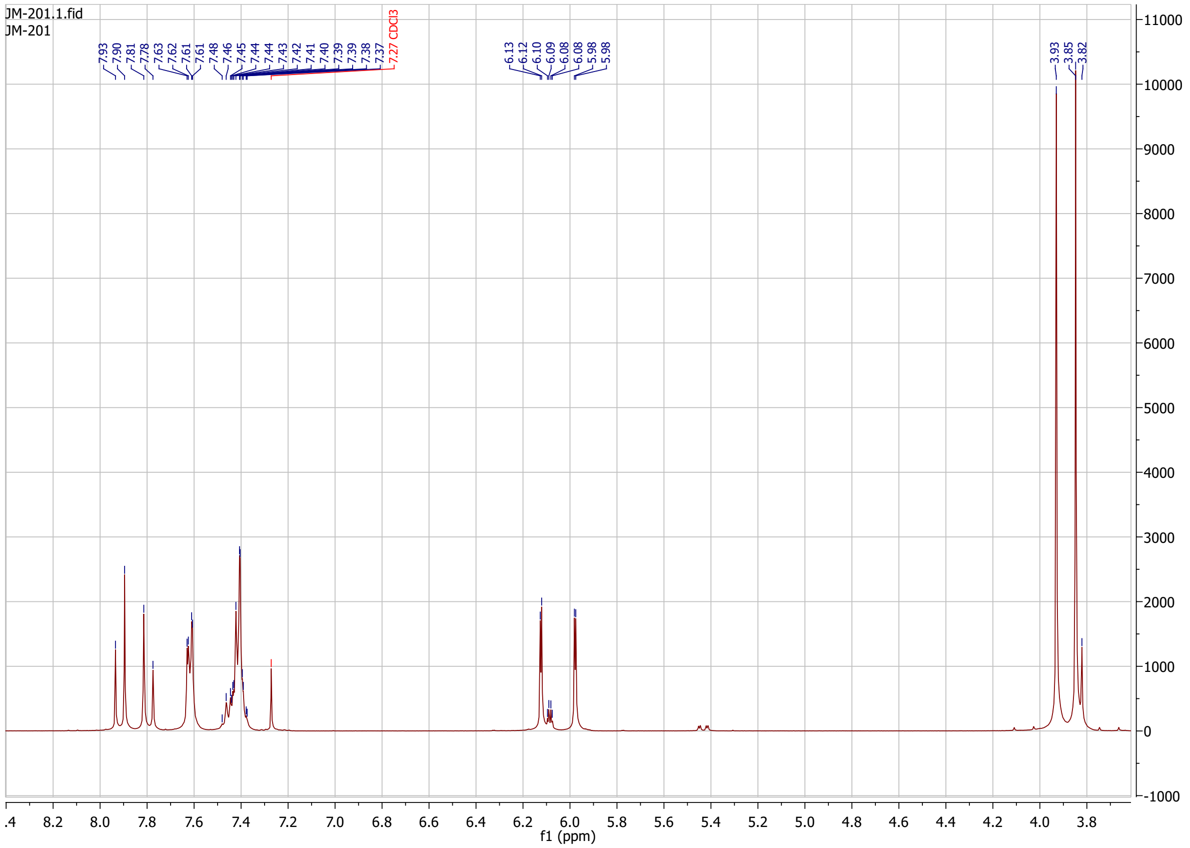

SI 3: ^{1}H NMR spectrum of compound **2** (500 MHz, $CDCl_3$)

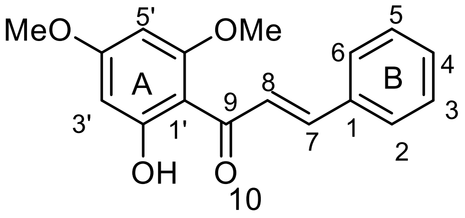

2

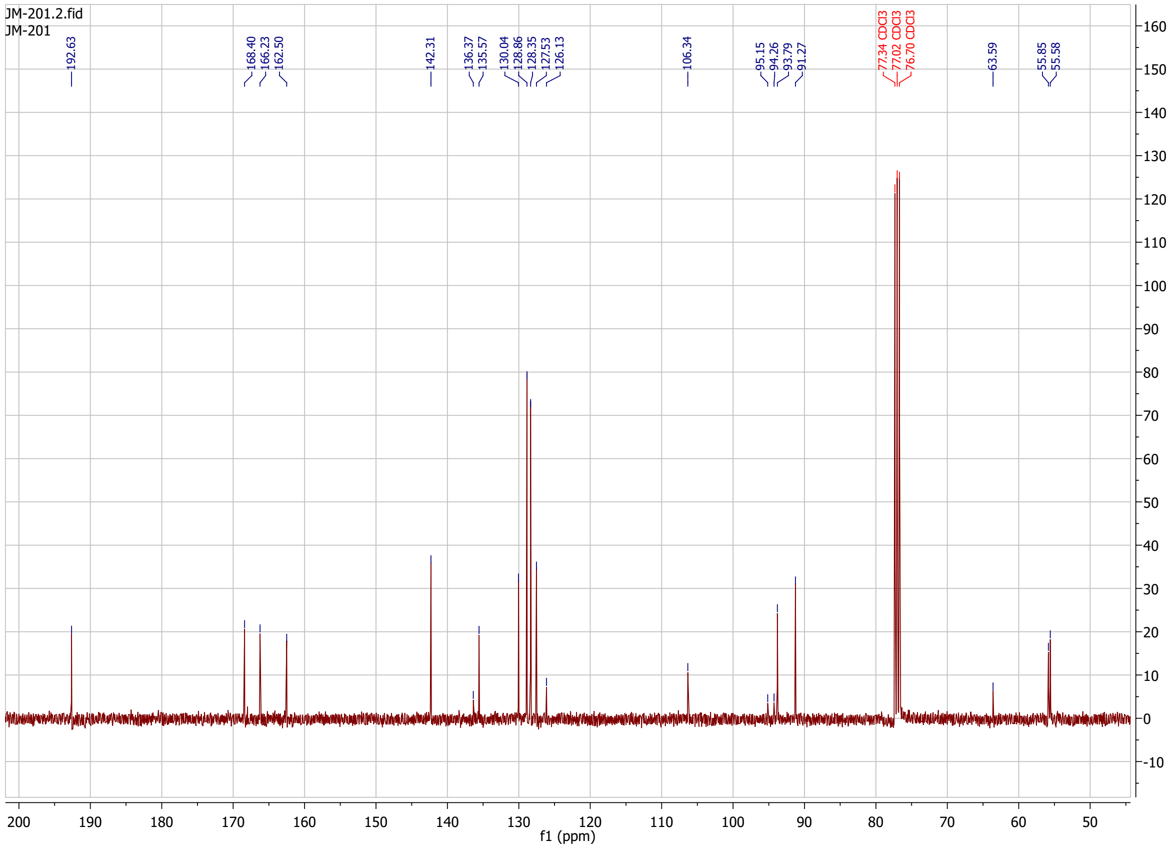

SI 4: ^{13}C NMR spectrum of compound **2** (125 MHz, $CDCl_3$)

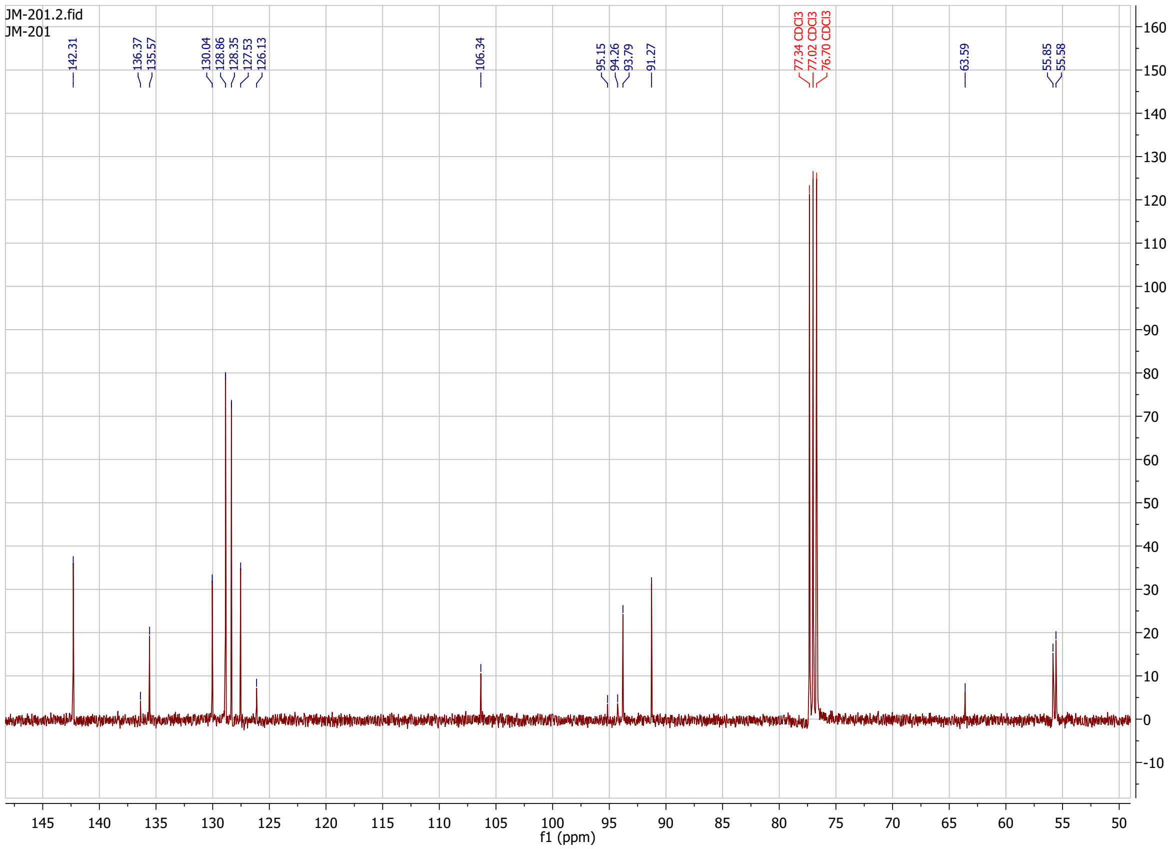

SI 4: ${}^{13}C$ NMR spectrum of compound **2** (125 MHz, $CDCl_3$)

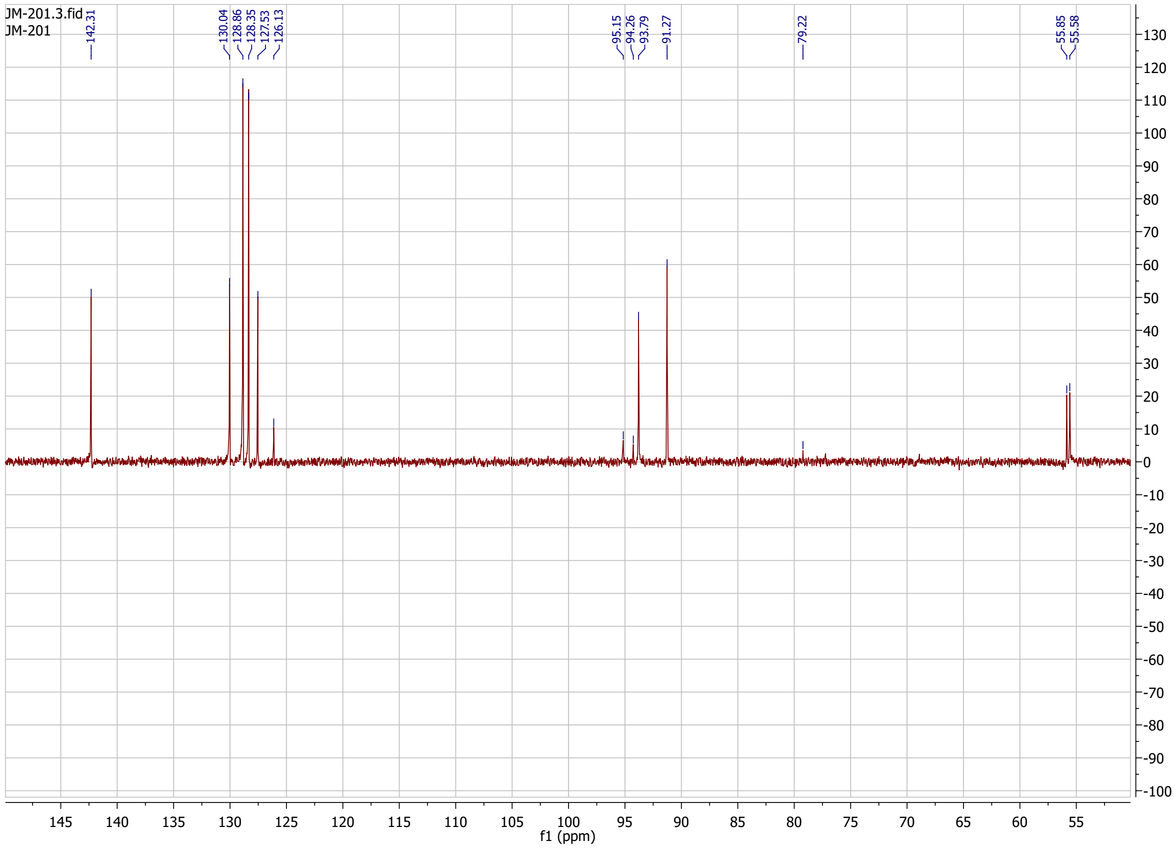

SI 4: ^{13}C NMR DEPT spectrum of compound **2** (125 MHz, $CDCl_3$)

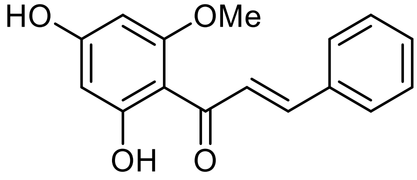

3

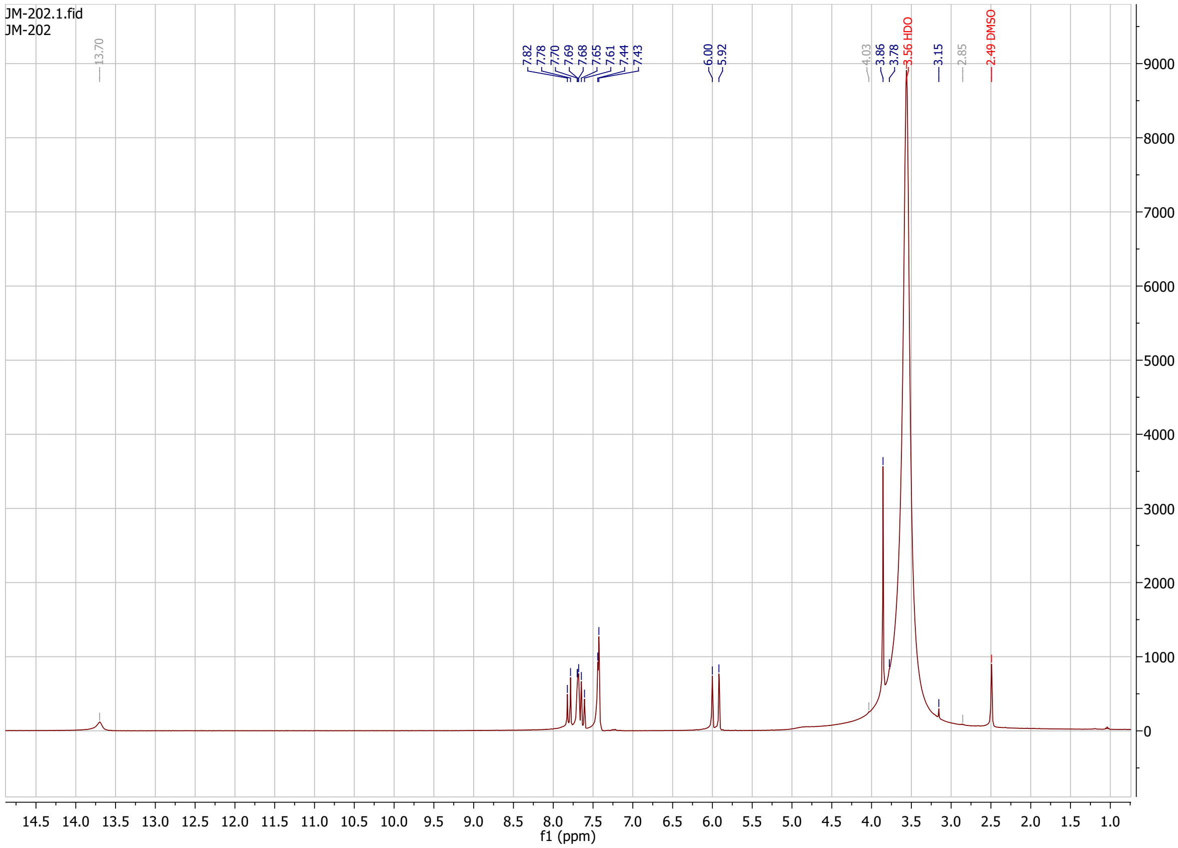

SI 5: ^{1}H NMR spectrum of compound **3** (500 MHz, CD_3OD)

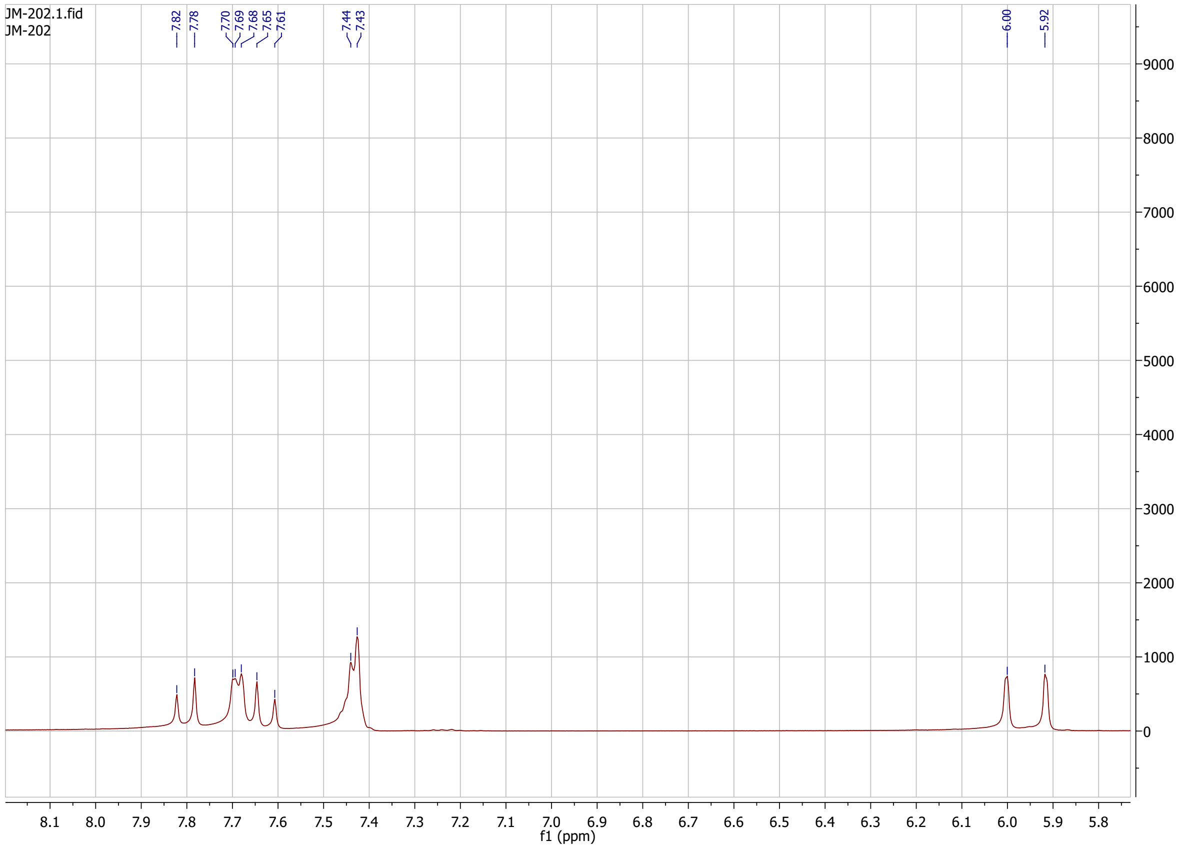

SI 5: ^{1}H NMR spectrum of compound **3** (500 MHz, CD_3OD)

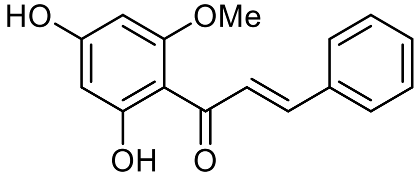

3

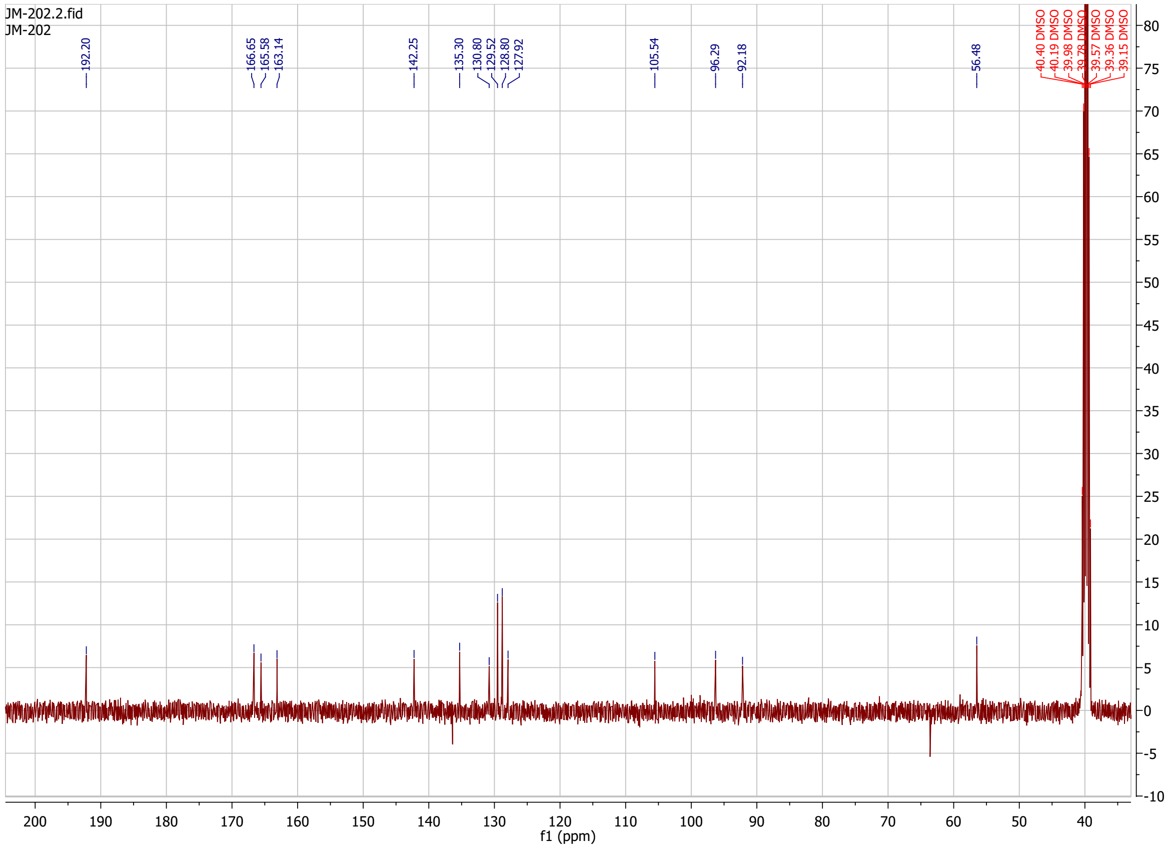

SI 6: ^{13}C NMR spectrum of compound **3** (125 MHz, CD_3OD)

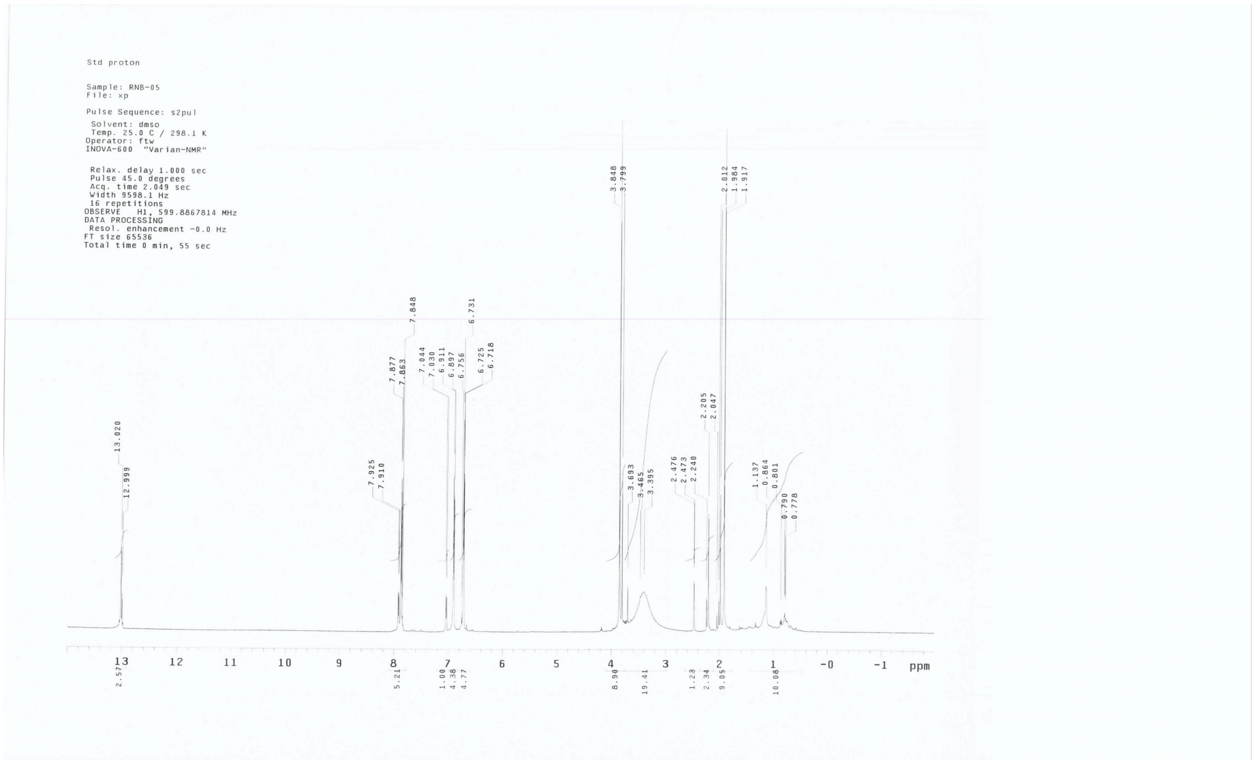

SI 7: ^{1}H NMR spectrum of compound **4** (500 MHz, CD_3OD)

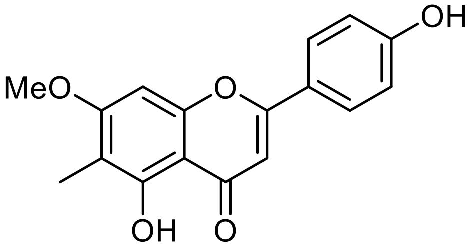

4

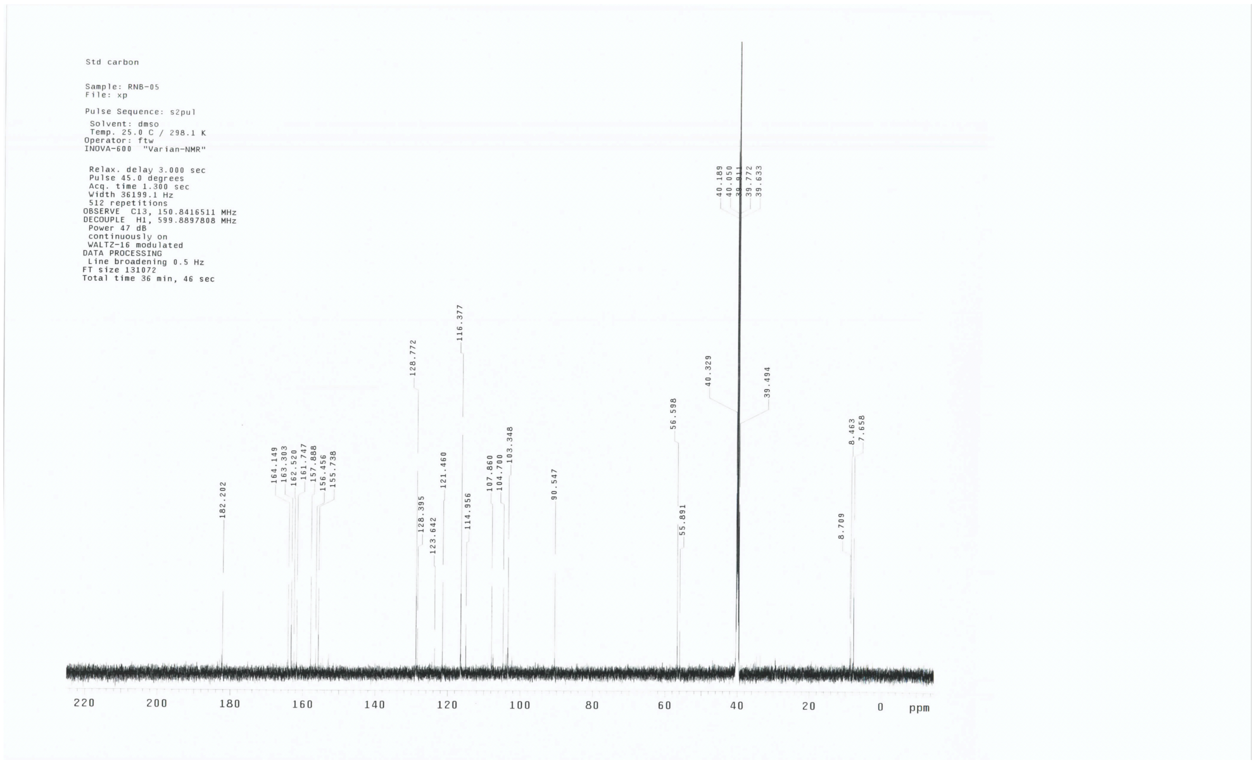

SI 8: ^{13}C NMR spectrum of compound **4** (125 MHz, CD_3OD)

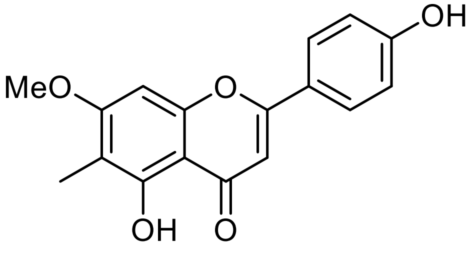

4

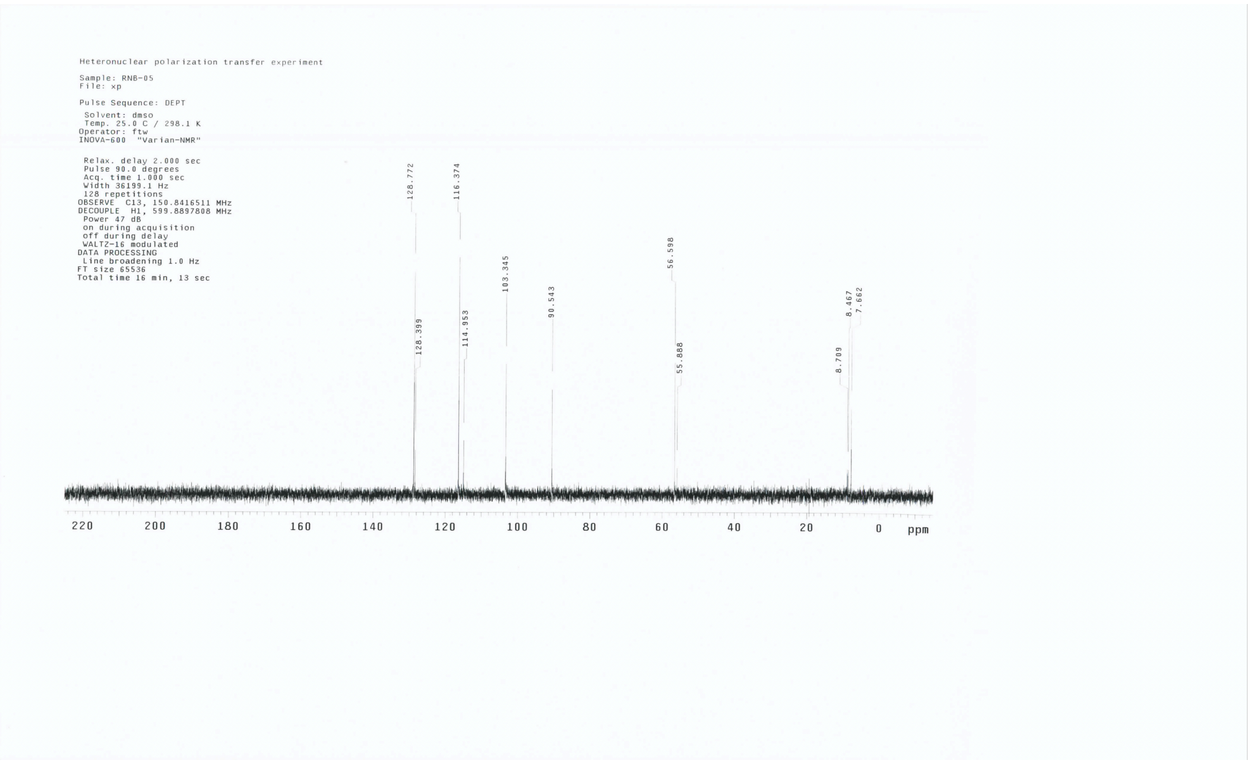

SI 8: ^{13}C NMR spectrum of compound **4** (125 MHz, CD_3OD)

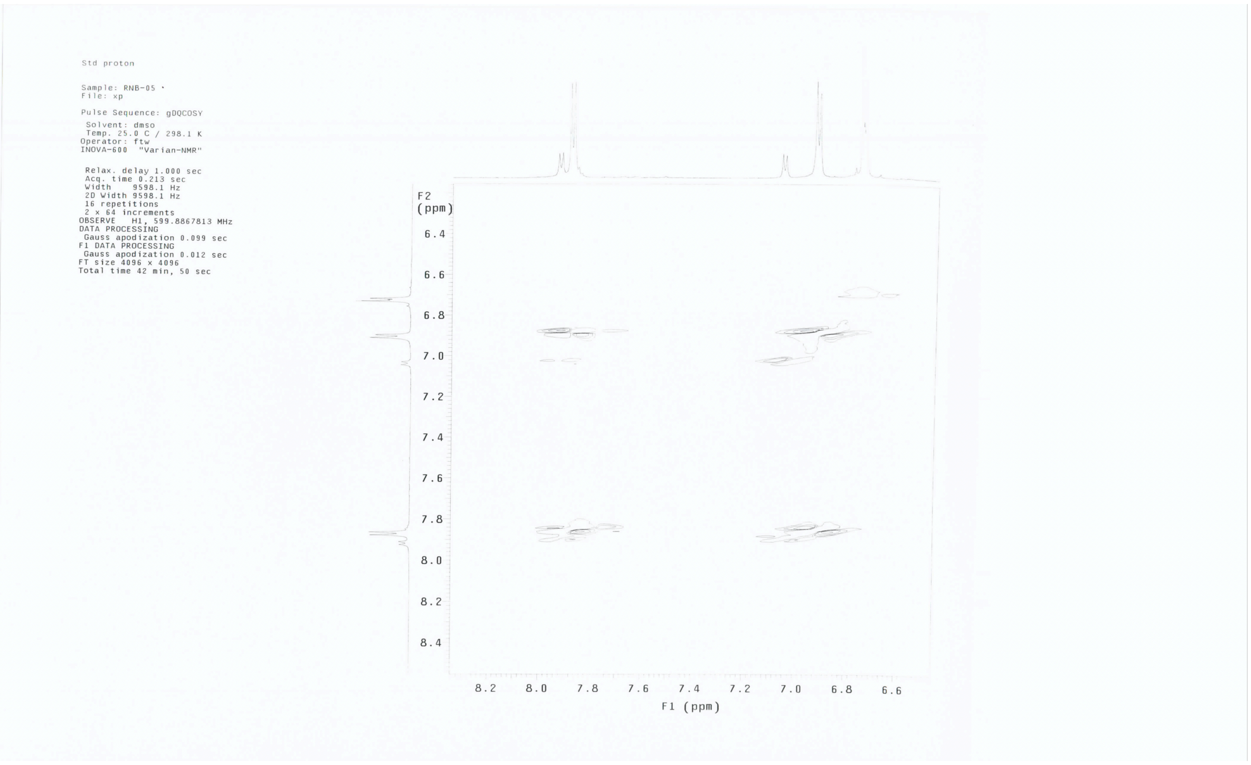

SI 9: 2D- NMR COSY spectrum of compound **4** (125 MHz, CD_3OD)

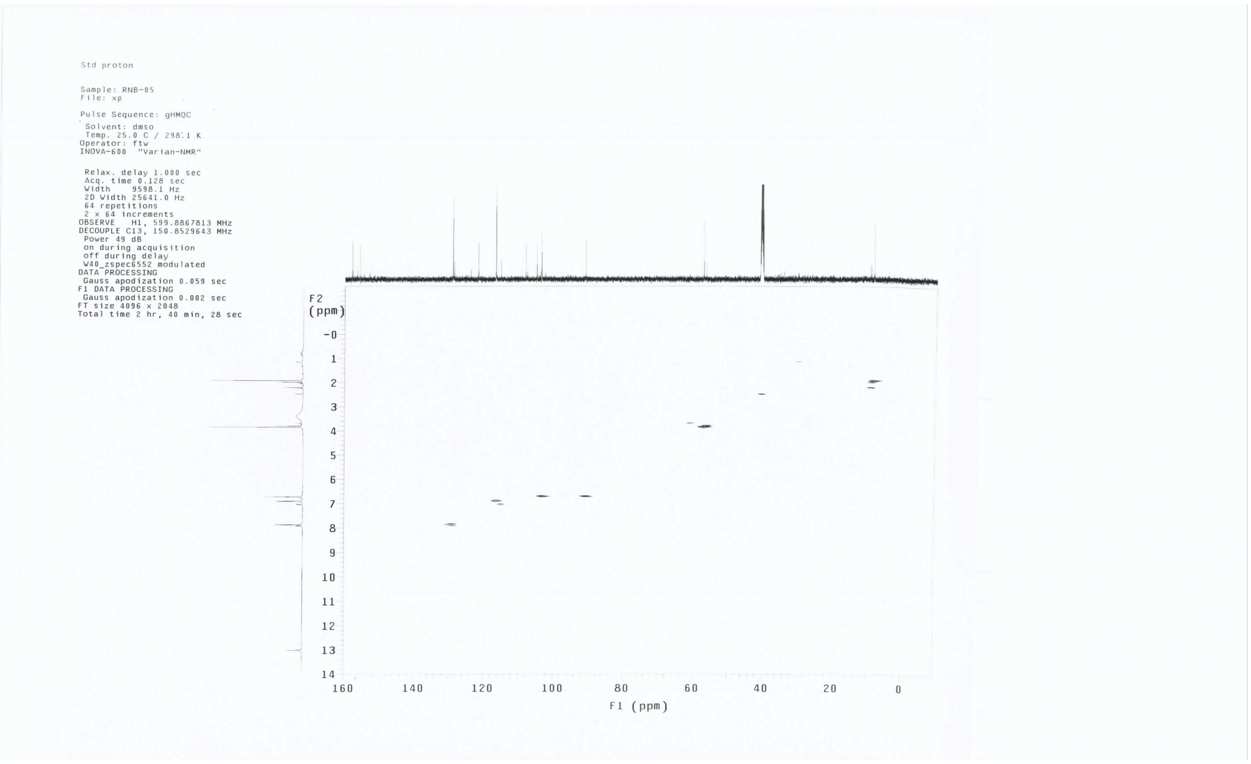

SI 10: 2D- NMR HMQC spectrum of compound **4** (500 MHz, CD_3OD)

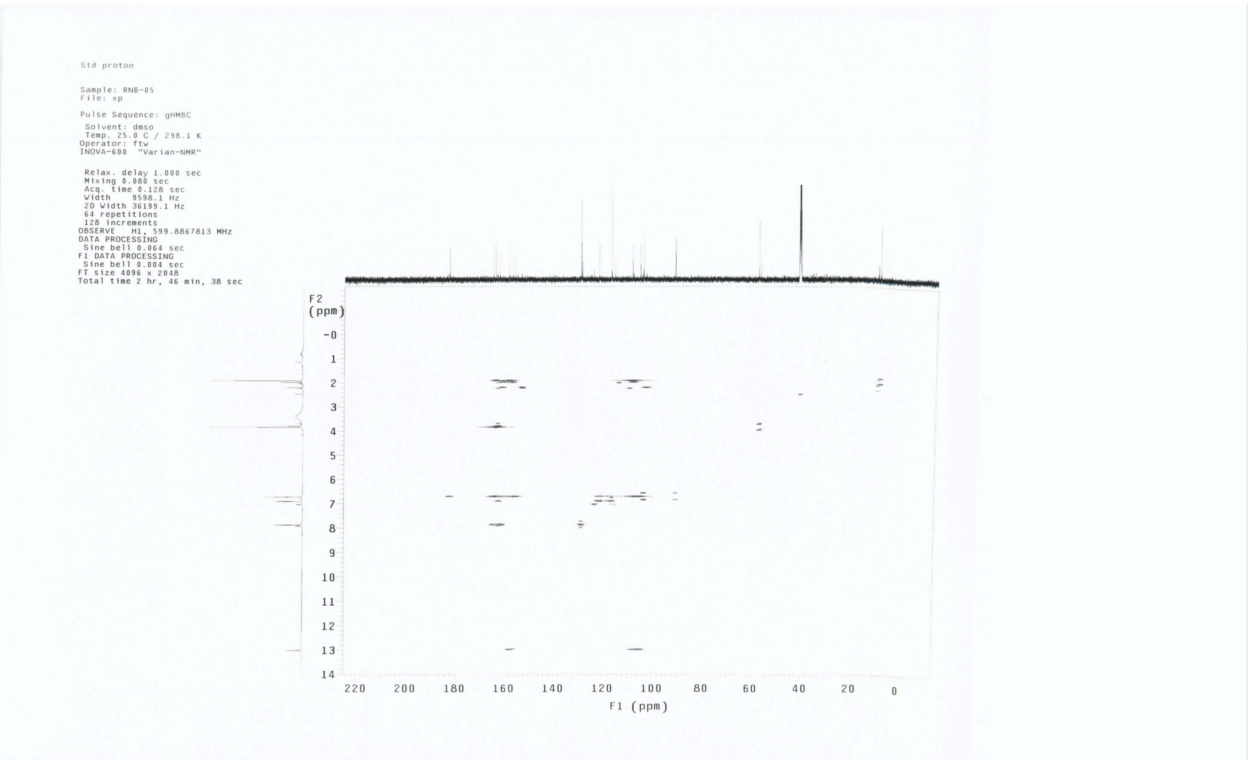

SI 11: 2D- NMR HMBC spectrum of compound **4** (500 MHz, CD_3OD)

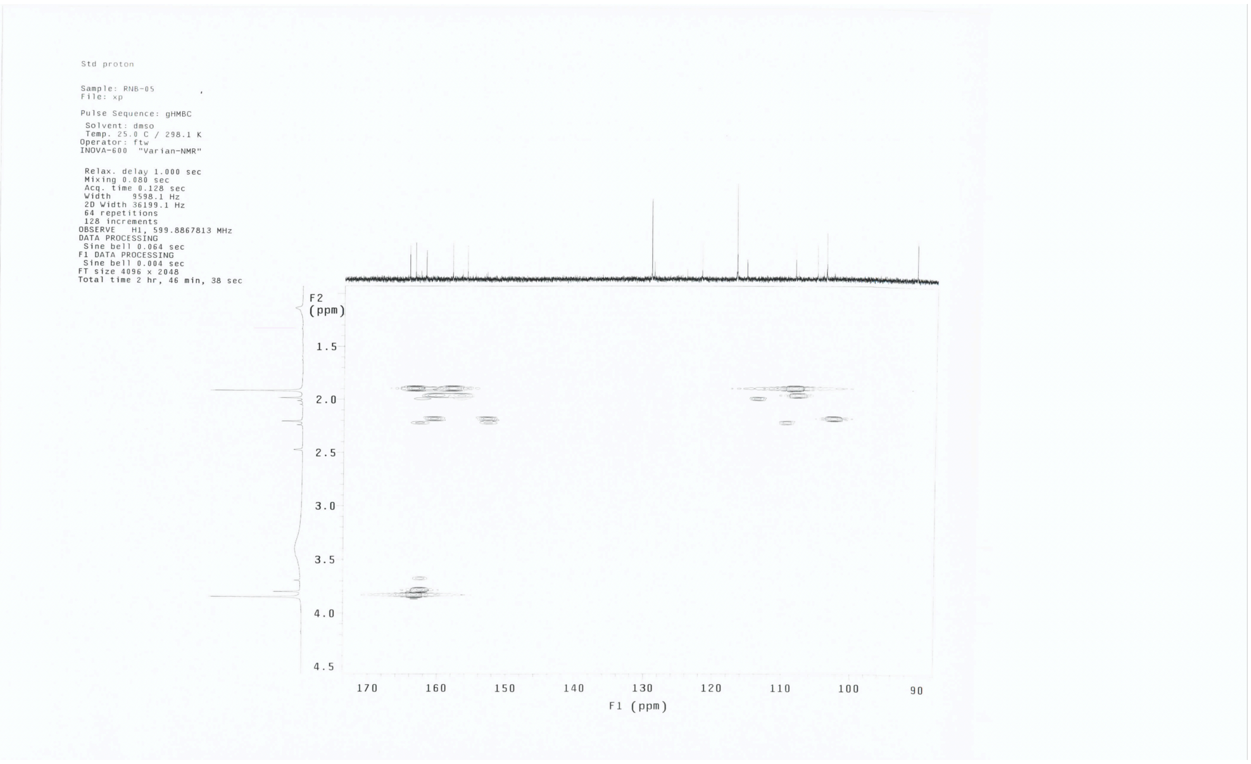

SI 11: 2D- NMR HMQC spectrum of compound **4** (500 MHz, CD_3OD)

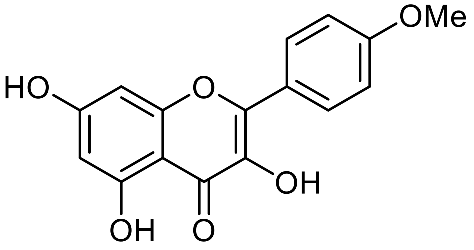

5

EOA-35P, 1H-NMR, DMSO, Varian Mercury-400

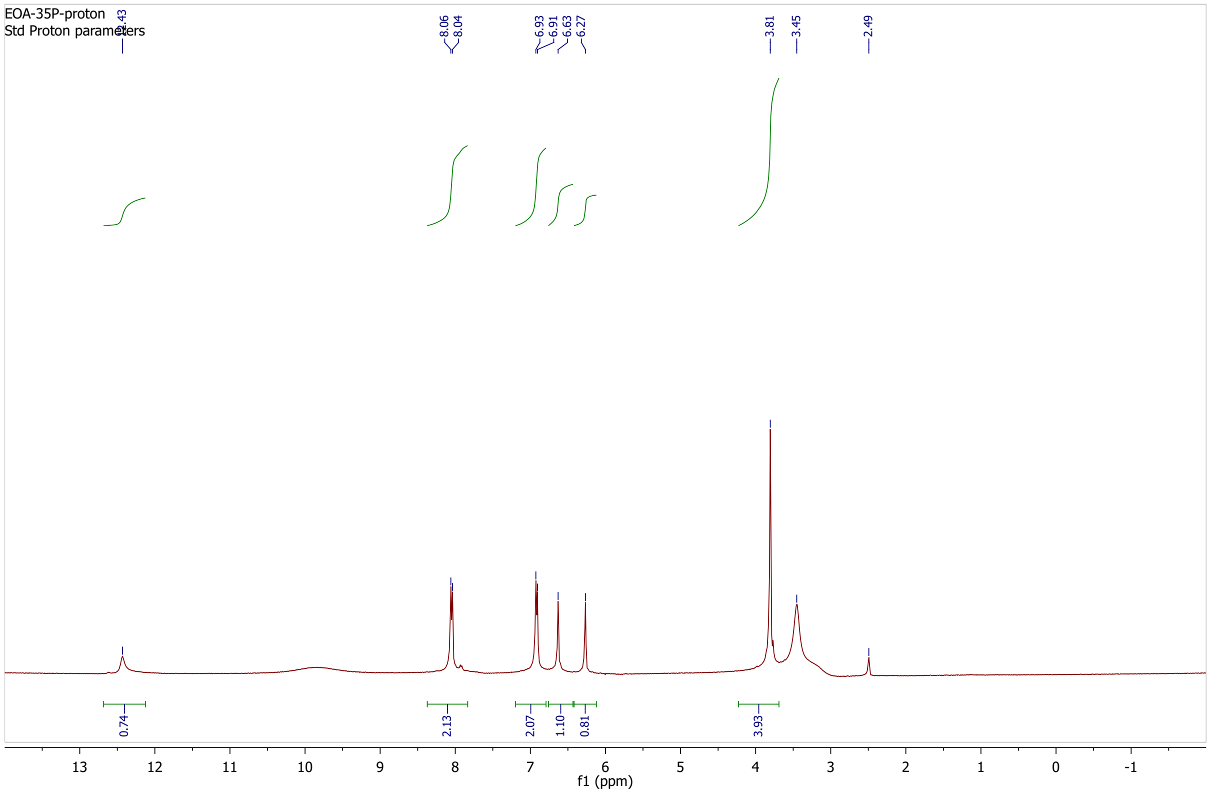

SI 12: ^{1}H NMR spectrum of compound **5** (500 MHz, CD_3OD)

EOA-35P, 1H-NMR, DMSO, Varian Mercury-400

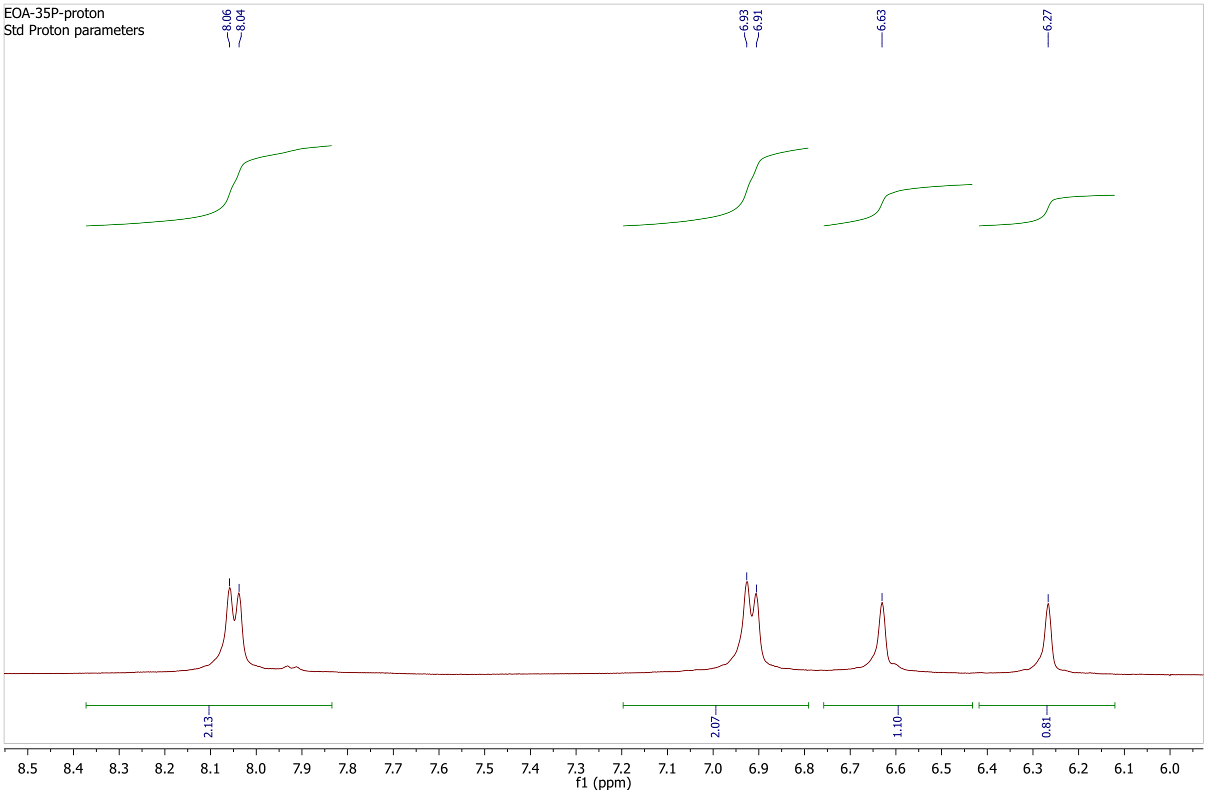

SI 12: ^{1}H NMR spectrum of compound **5** (500 MHz, CD_3OD)

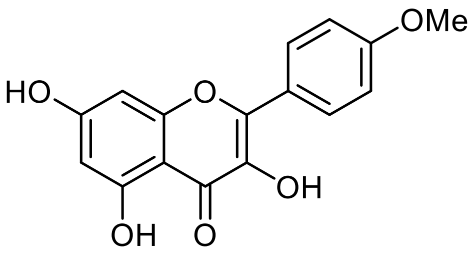

5

EOA-35P, 13C-NMR, DMSO, Varian Mercury-400

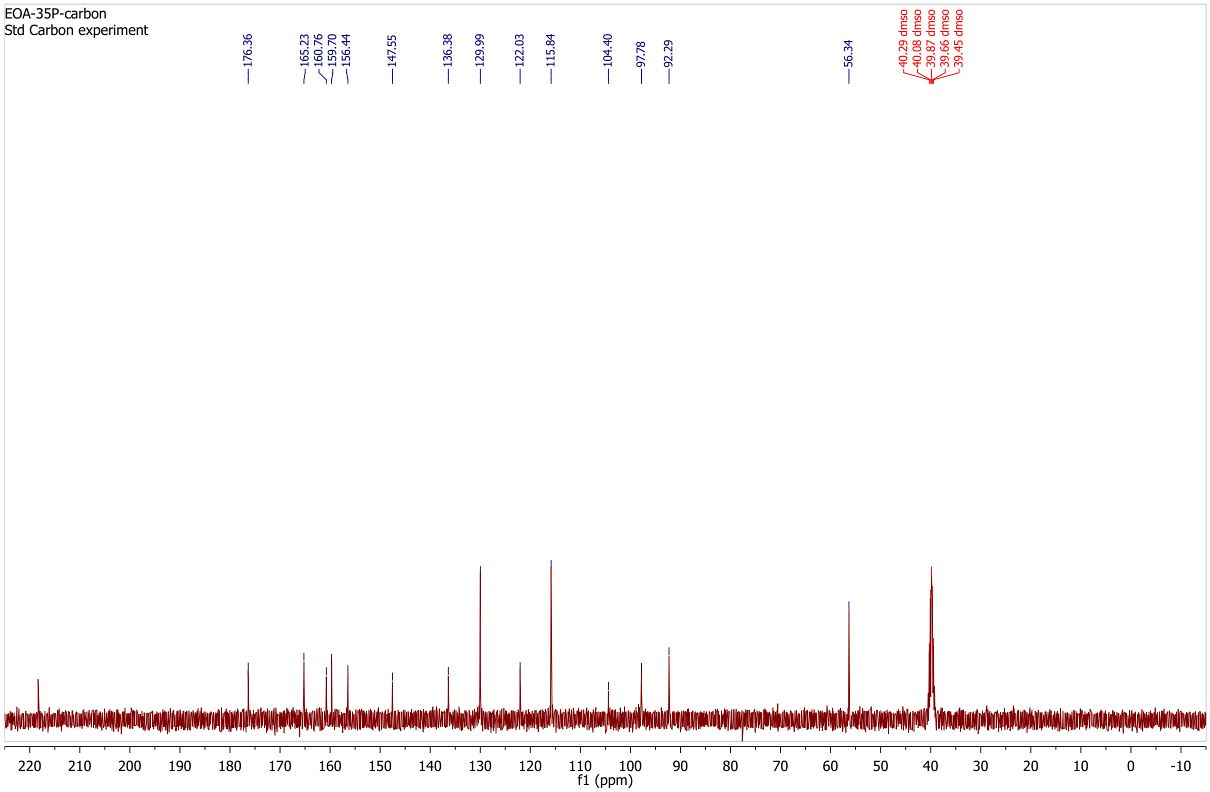

SI 13: ^{13}C NMR spectrum of compound **5** (125 MHz, CD_3OD)

EOA-35P, DEPT-135, DMSO, Varian Mercury-400

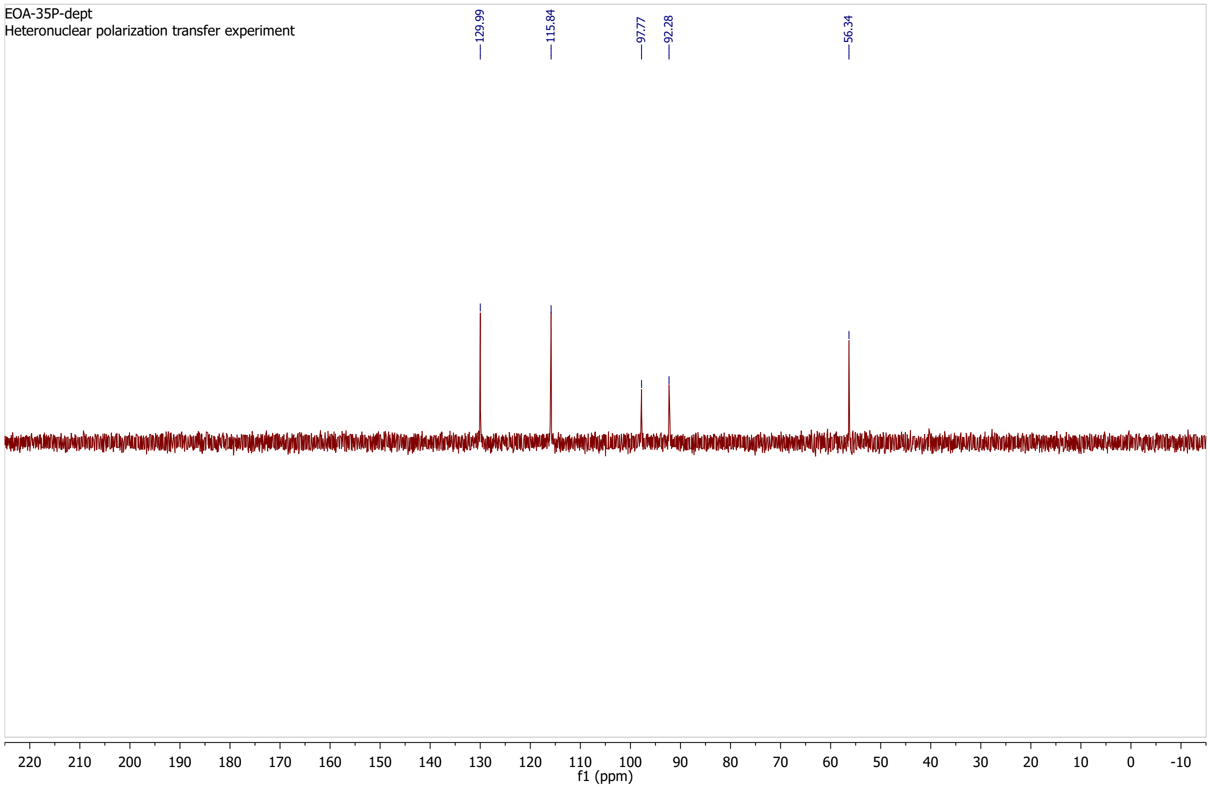

SI 13: ^{13}C NMR DEPT spectrum of compound **5** (125 MHz, CD_3OD)

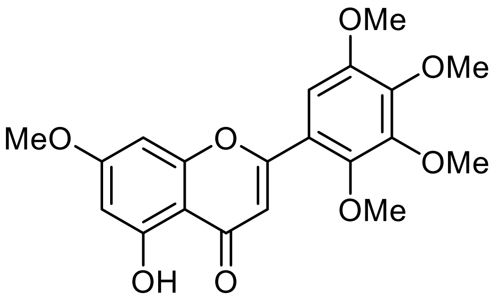

6

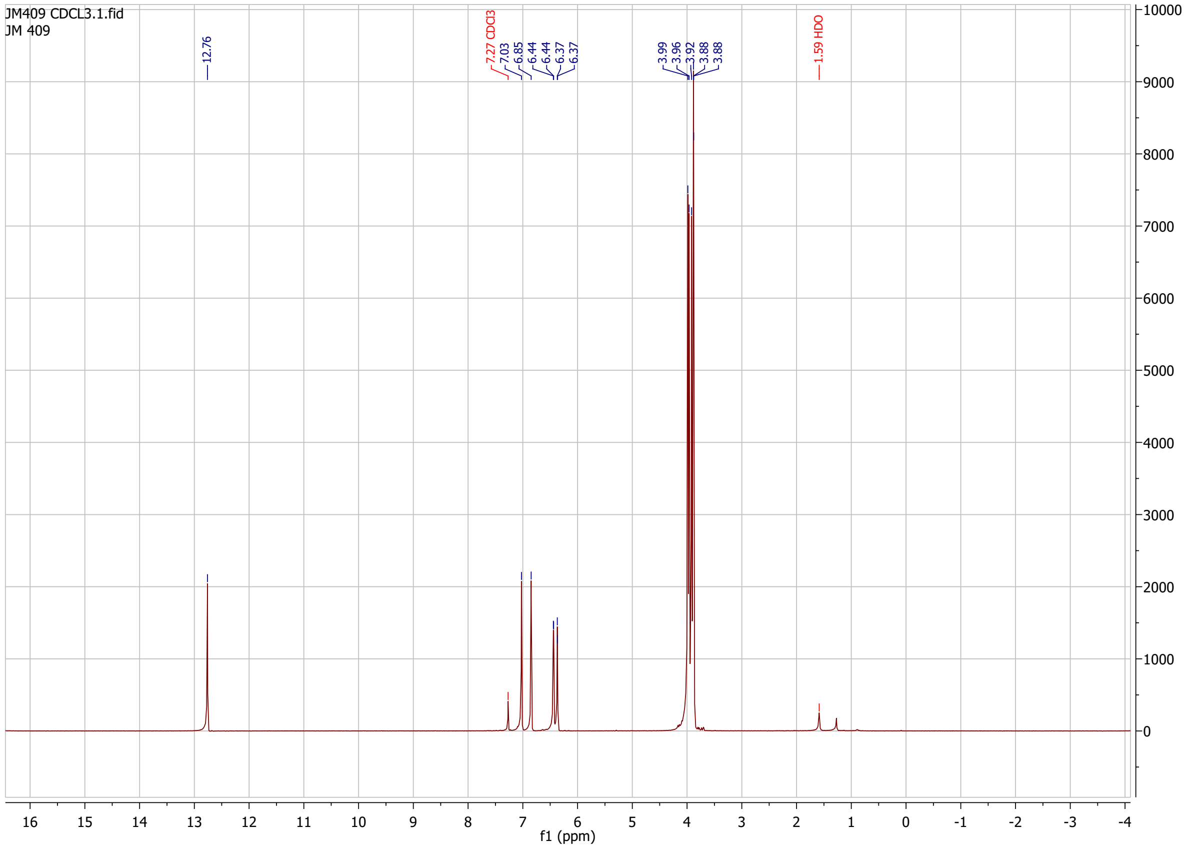

SI 14: ^{1}H NMR spectrum of compound **6** (500 MHz, $CHCl_3$+CD_3OD)

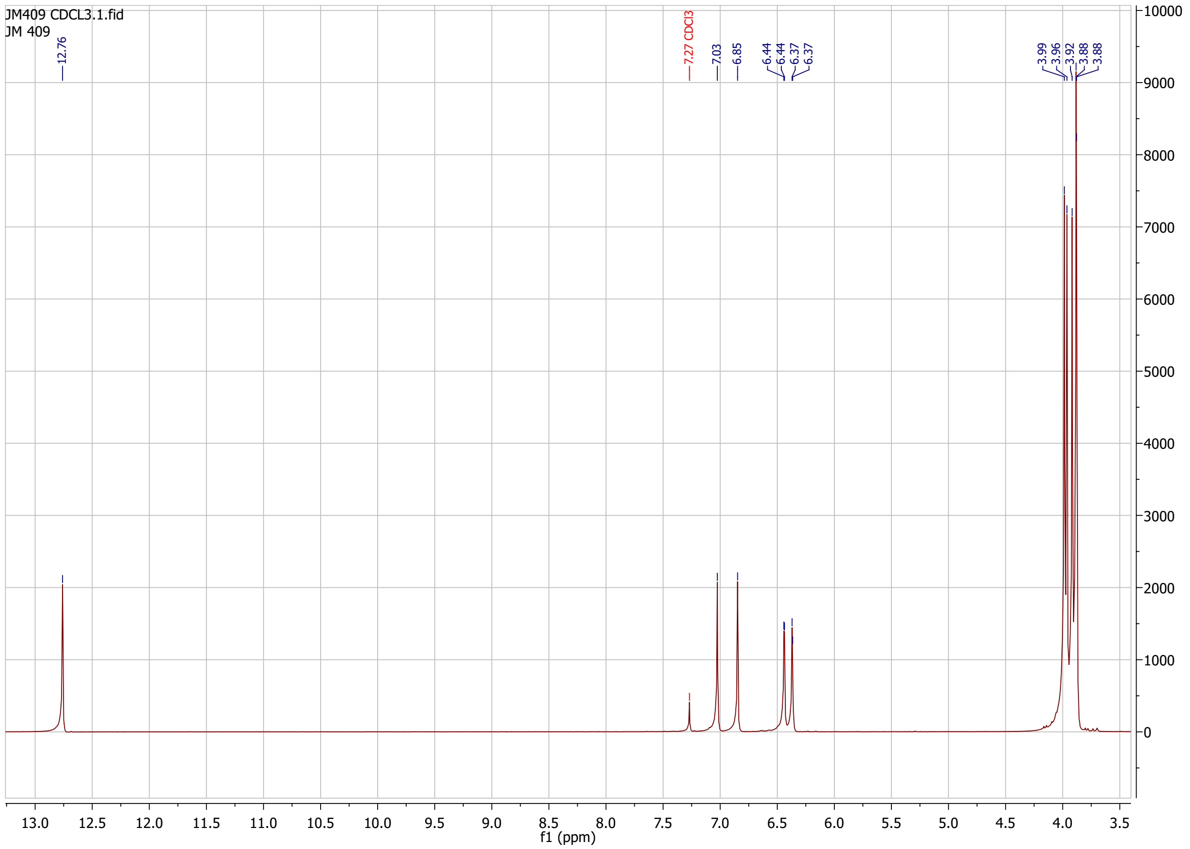

SI 14: ^{1}H NMR spectrum of compound **6** (500 MHz, $CHCl_3$+CD_3OD)

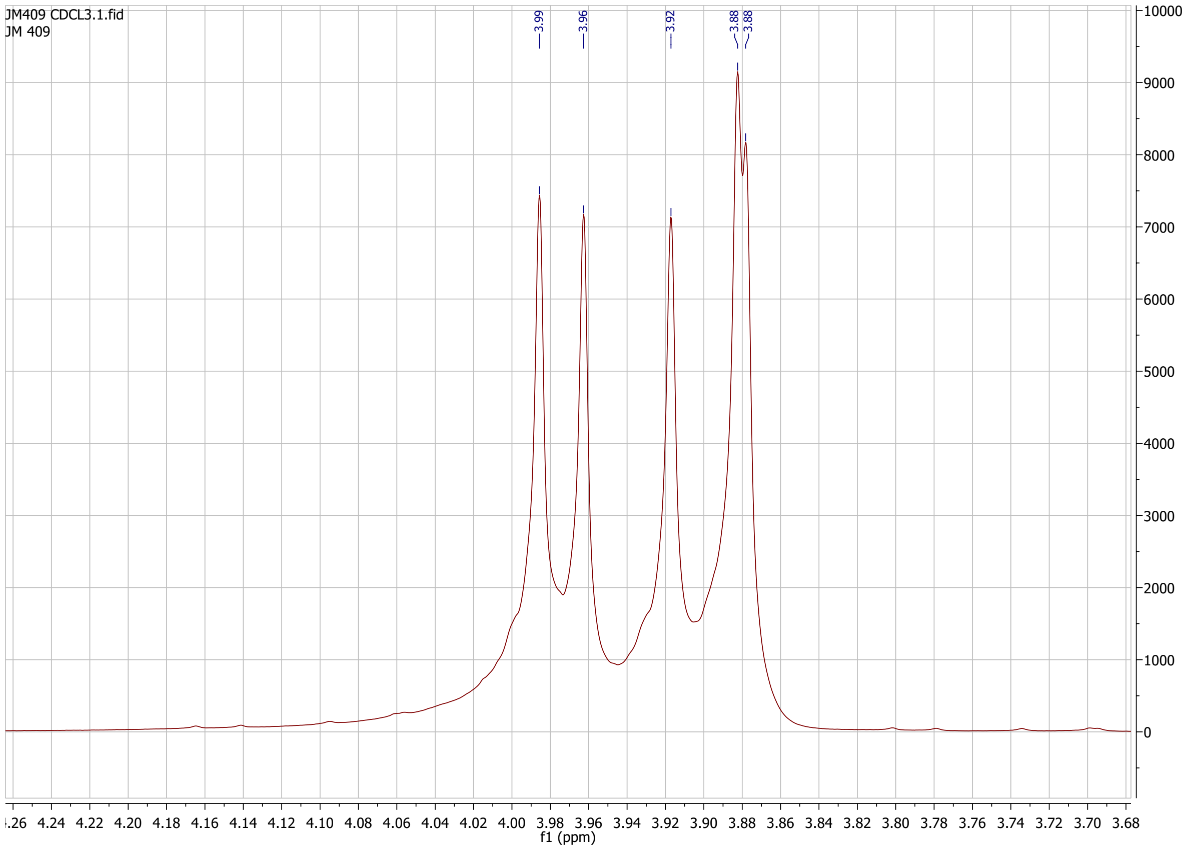

SI 14: ^{1}H NMR spectrum of compound **6** (500 MHz, $CHCl_3$+CD_3OD)

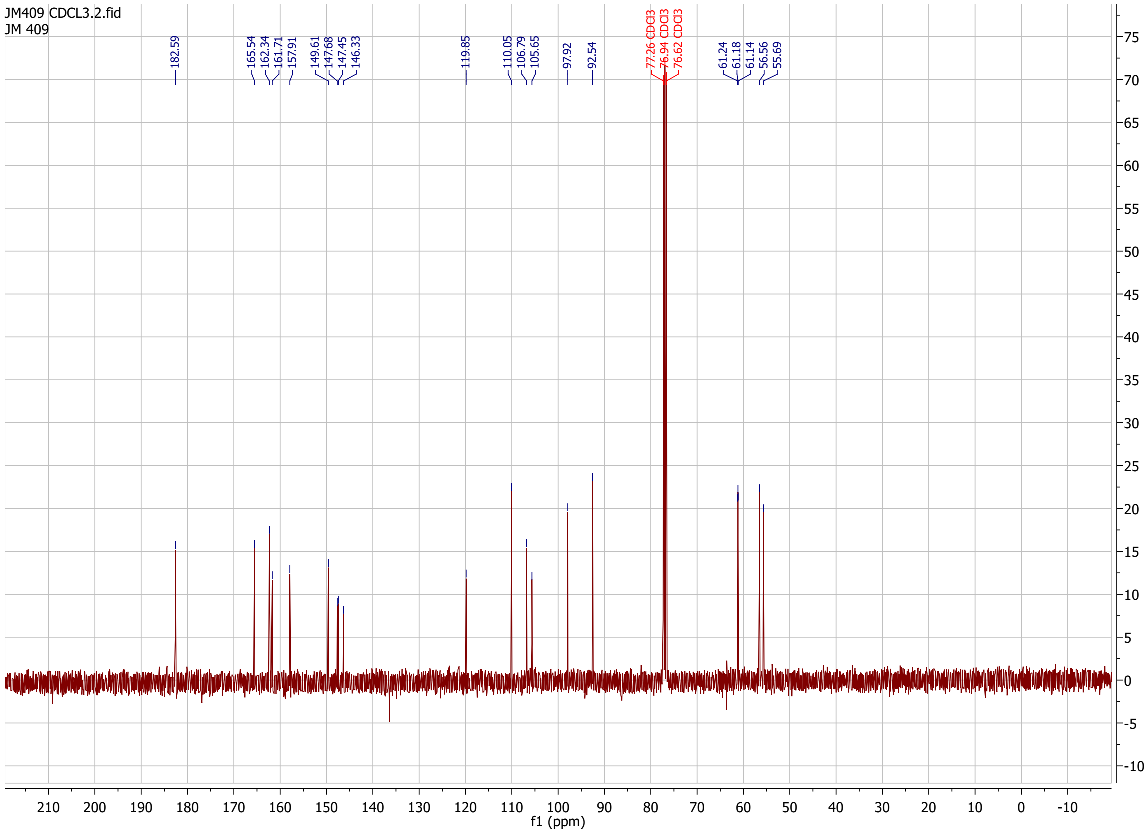

JM409 CDCL3.2.fid
JM 409
182.59
165.54
162.34
161.71
157.91
149.61
147.68
147.45
146.33
119.85
110.05
106.79
105.65
97.92
92.54
77.26 CDCl3
76.94 CDCl3
76.62 CDCl3
61.24
61.18
61.14
56.56
55.69
f1 (ppm)

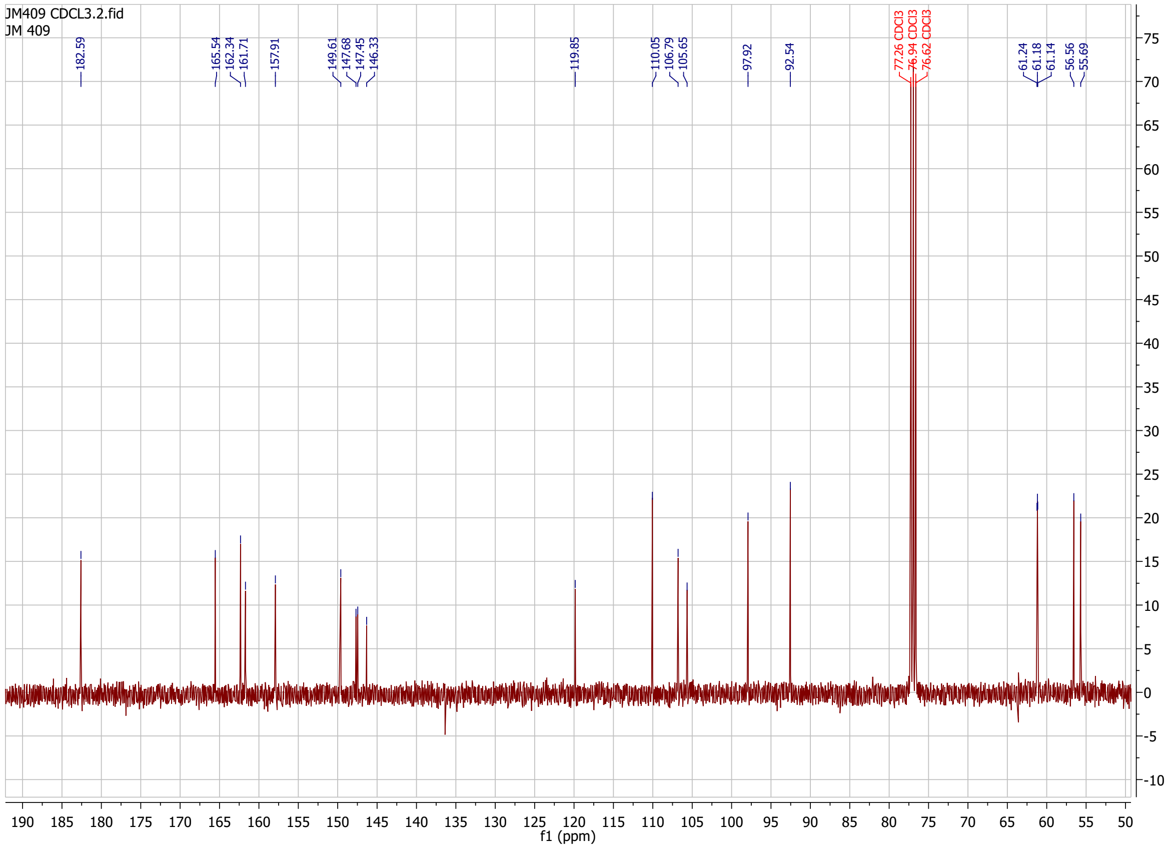

SI 15: ^{1}H NMR spectrum of compound **6** (500 MHz, $CHCl_3$+CD_3OD)

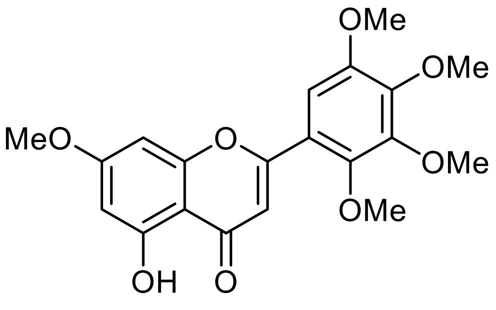

6

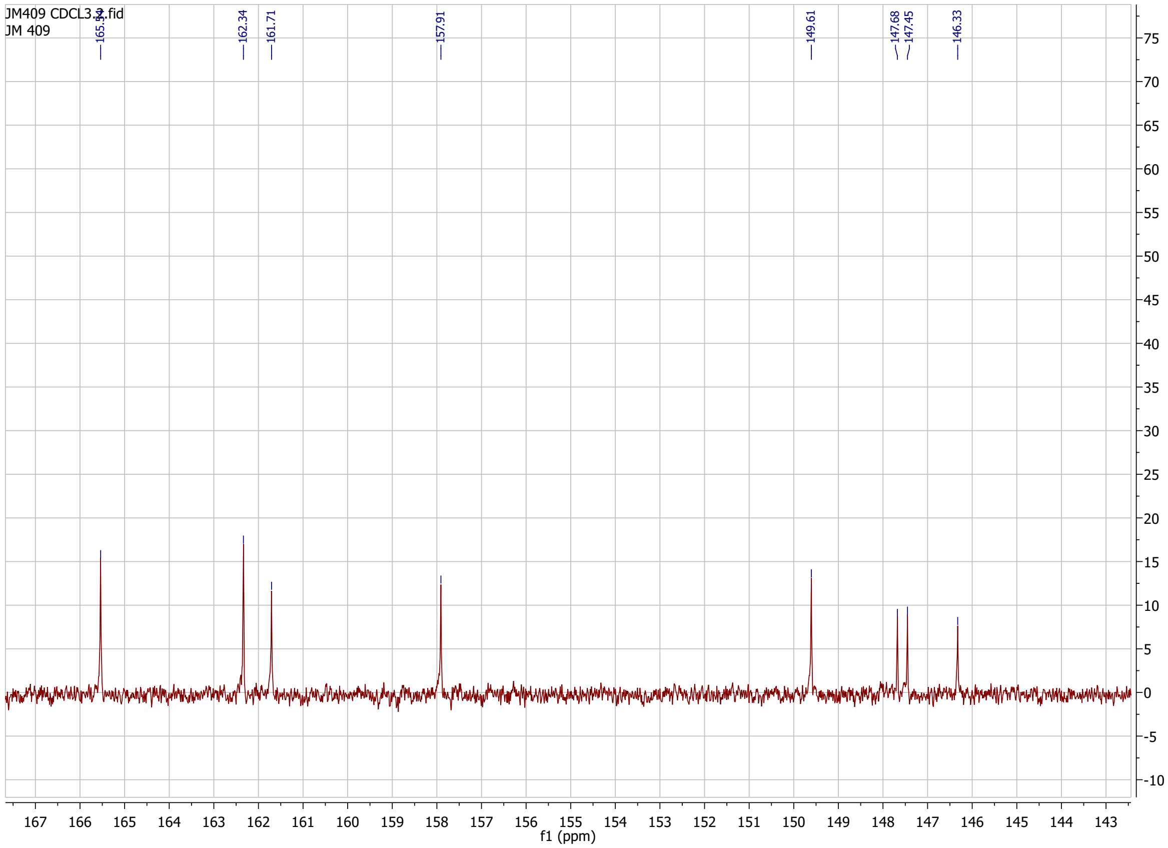

SI 15: ^{1}H NMR spectrum of compound **6** (500 MHz, $CHCl_3$+CD_3OD)

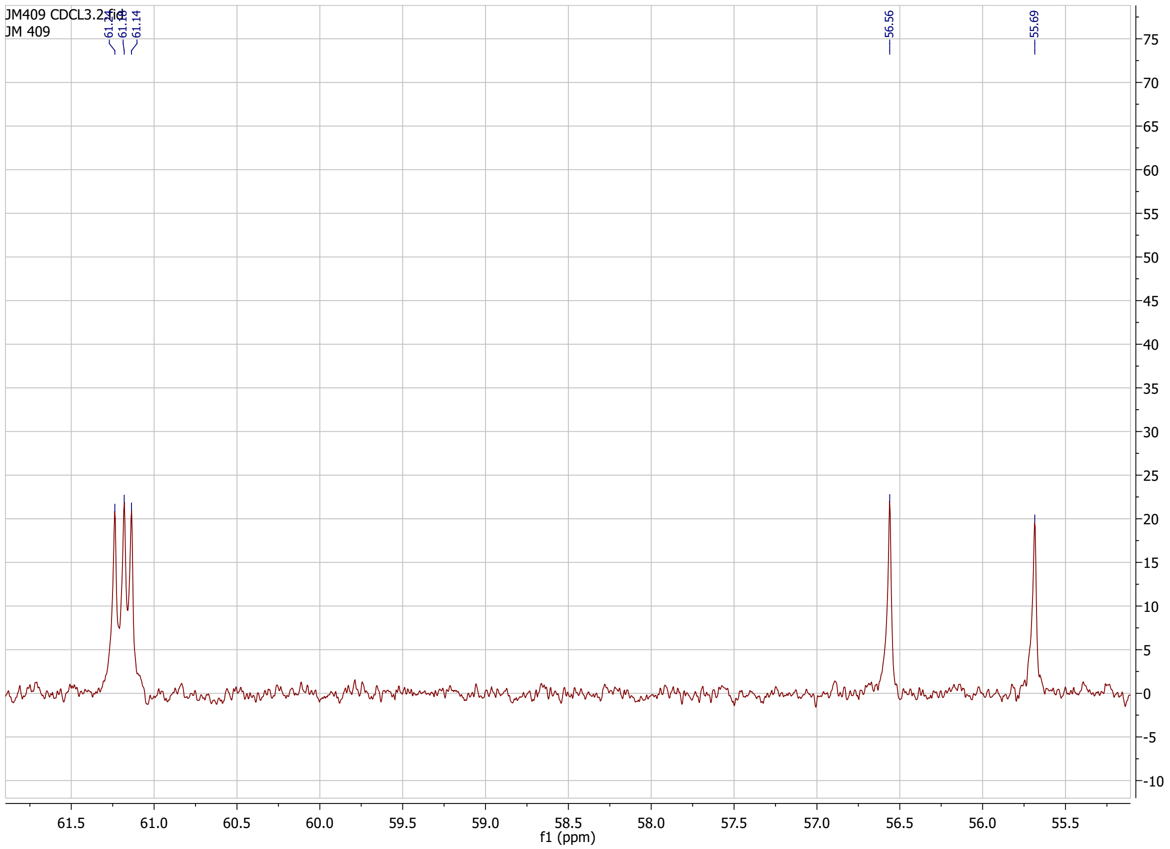

SI 15: ^{13}C NMR spectrum of compound **6** (125 MHz, $CHCl_3$+CD_3OD)
